# Oncologic outcome of marginal mandibulectomy in squamous cell carcinoma of the lower gingiva

**DOI:** 10.1186/s12885-019-5999-0

**Published:** 2019-08-06

**Authors:** Wei Du, Qigen Fang, Yao Wu, Junfu Wu, Xu Zhang

**Affiliations:** Department of Head Neck and Thyroid, Affiliated Cancer Hospital of Zhengzhou University, Henan Cancer Hospital, Zhengzhou, Henan Province People’s Republic of China

**Keywords:** Gingiva squamous cell carcinoma, Oral squamous cell carcinoma, Marginal mandibulectomy, Prognosis

## Abstract

**Background:**

There is a large amount of controversy about the best management of the mandible in oral squamous cell carcinoma (SCC), mainly owing to the inability to acquire accurate bone invasion status. Therefore, our goal was to analyse the oncologic safety in patients undergoing marginal mandibulectomy (MM) for cT1-2 N0 SCC of the lower gingiva.

**Methods:**

Patients undergoing MM for untreated cT1-2 N0 SCC of the lower gingiva were retrospectively enrolled. The main endpoints of interest were locoregional control (LRC) and disease-specific survival (DSS).

**Results:**

A total of 142 patients were included in the analysis, and a pathologic positive node was noted in 27 patients. Cortical invasion was reported in 23 patients, and medullary invasion was reported in 9 patients. The 5-year LRC and DSS rates were 85 and 88%, respectively. Patients with bone invasion had a significantly higher risk for recurrence than patients without bone invasion. However, the DSS was similar in patients with versus without bone invasion. Patients with a high neutrophil lymphocyte ratio had a higher risk for worse prognosis.

**Conclusions:**

The oncologic outcome in patients undergoing MM for cT1-2 N0 SCC of the lower gingiva was favourable; bone invasion was not uncommon, but it significantly decreased the prognosis in patients undergoing MM.

## Background

There is a large amount of controversy about the best management of the mandible in oral squamous cell carcinoma (SCC), mainly owing to the inability to acquire accurate bone invasion status [1, 2]. Although adjuvant examinations help with decision making during treatment of the mandible, negative radiological presentation does not completely eliminate the possibility of bone invasion, especially in early stage oral cancer.

The effect of bone invasion on prognosis has been widely analysed. O’Brien et al. [3] described that histological bone invasion rates were 64 and 16% in segmental and marginal groups, respectively. Moreover, the authors concluded that local recurrence was mainly attributed to positive soft tissue margins but not the mandible resection method. Similarly, Tei et al. [4] reported a higher bone invasion rate in the segmental group, but it did not translate into a survival difference. Both studies suggested that unless there was a positive soft tissue margin, marginal mandibulectomy (MM) was a safe procedure for selected oral cancer patients.

Oncologic outcome after MM for oral SCC has rarely been analysed. Werning et al. [5] reported that the overall local and regional recurrence and distant metastasis rate for all stages were 14.4, 18.0, and 2.7%, respectively. A total of 69.8% of the patients remained alive without disease 2 years after treatment. Petrovic et al. [6] reported that after a follow-up of a mean time of 55.1 months, 67 and 39 patients developed local and regional recurrence, and the 5-year local control and regional control rates were 74.6 and 85.2%, respectively.

SCC of the lower gingiva is uncommon, and MM might be most likely to be performed for selected patients with gingiva SCC, but its prognosis still remains unclear. Therefore, in this study, we aimed to analyse the oncologic outcome in patients undergoing MM for cT1-2 N0 SCC of the lower gingiva.

## Methods

The Zhengzhou University institutional research committee approved our study (No. FHN2018087), and all participants signed an informed consent agreement for medical research before initial treatment. All methods were performed in accordance with relevant guidelines and regulations.

From January 1995 to January 2016, patients (≥18 years) undergoing MM for untreated cT1-2 N0 SCC of the lower gingiva were retrospectively enrolled. Patients without adequate follow-up information (at least 2 years) were excluded. Data regarding age, sex, TNM stage (AJCC 7th edition), operation record, pathology report, and follow-up were extracted and analysed. All pathologic sections were re-reviewed.

**Fig. 1:**
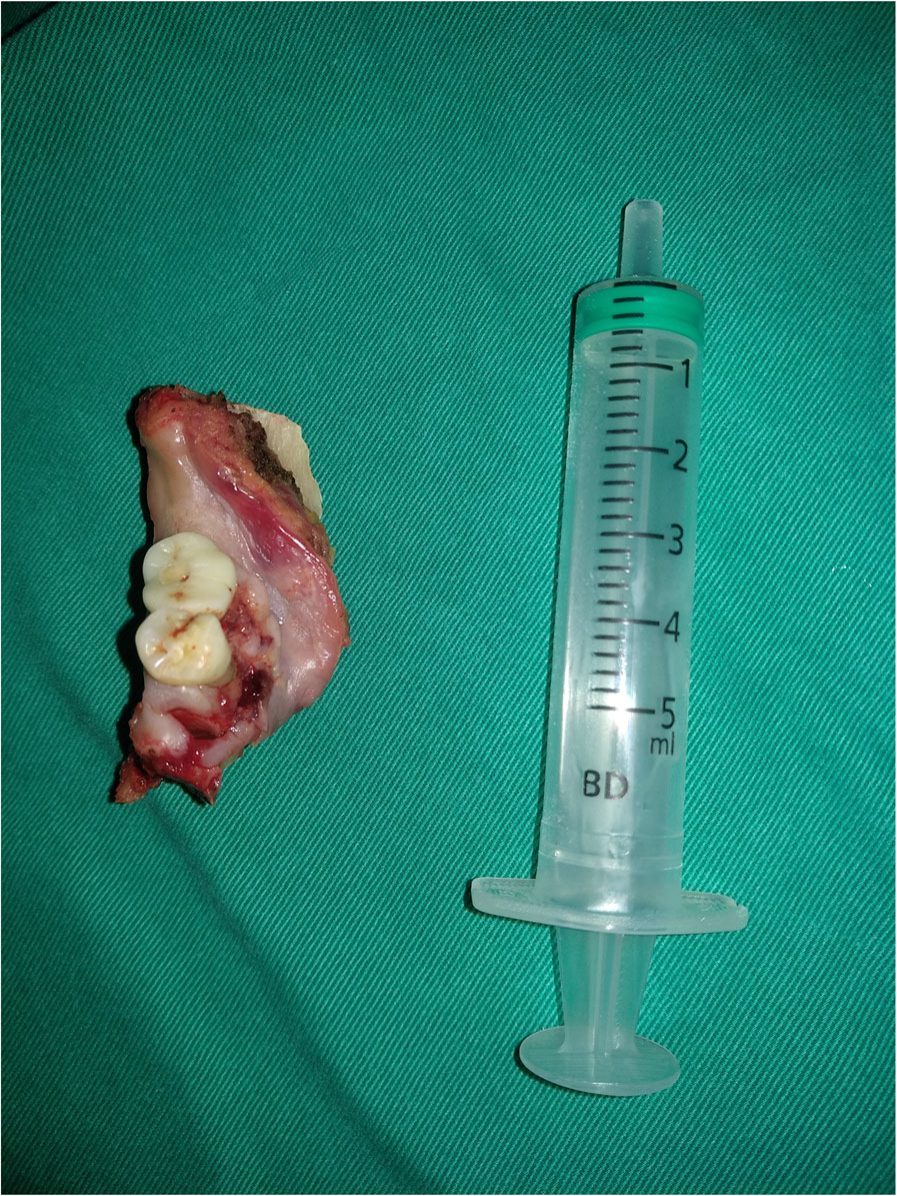
Stage cT1N0M0 squamous cell carcinoma of the lower gingiva

**Fig. 2:**
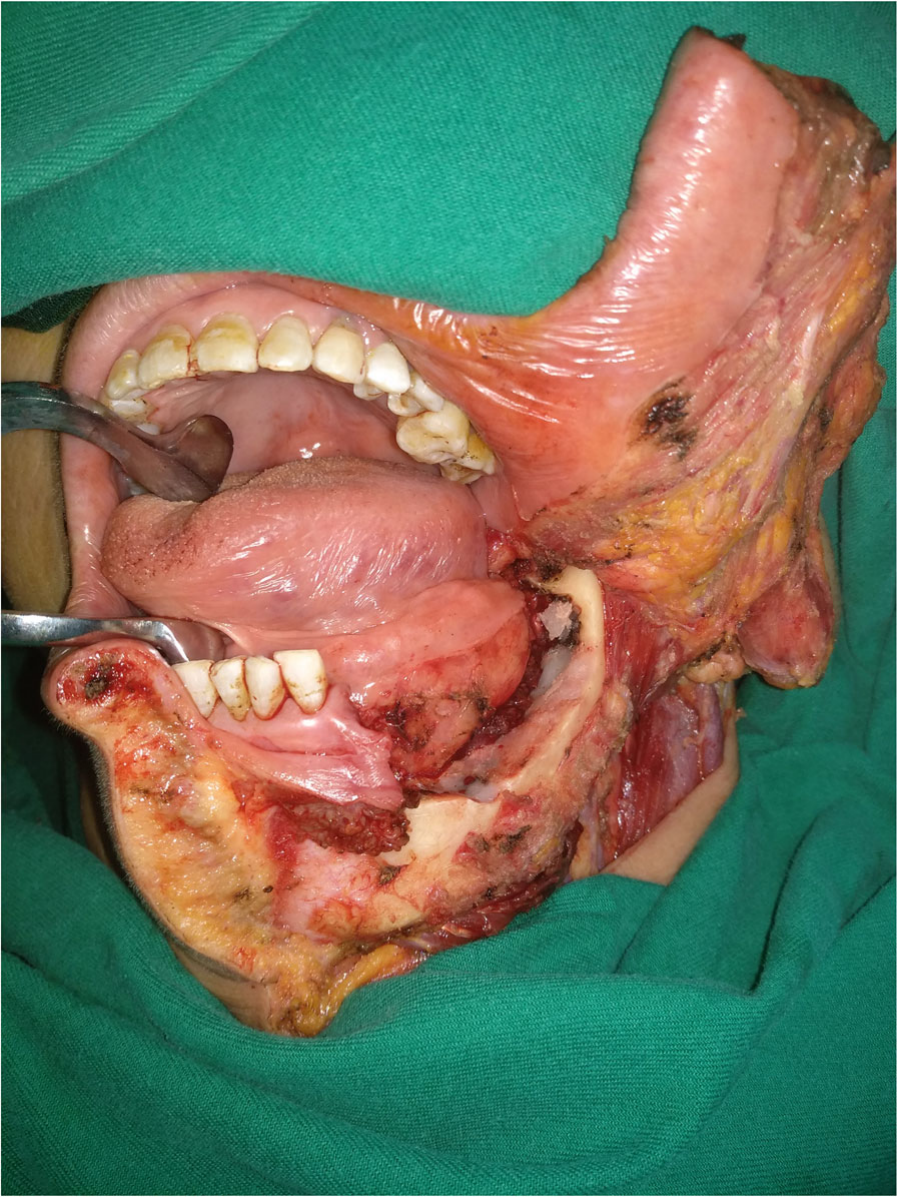
Marginal mandibulectomy: at least 10 mm of vertical height was preserved

In our cancer centre, MM is usually highly selected by the surgeons for patients with no or with minor bone invasion based on perioperative comprehensive consideration of clinical and imaging examination, intraoperative frozen sections (Fig. 1), tumour approximation and/or fixation of the underlying bony structure as well as the depth of the bony invasion. At least 10 mm of vertical height and of the mandibular canal were preserved to minimize the risk of pathological or iatrogenic fracture (Fig. 2). Neck dissection was performed for patients with SCC of the lower gingiva of any stage.

The main study endpoints were locoregional control (LRC) and disease-specific survival (DSS). The LRC survival time was calculated from the date of surgery to the date of first locoregional recurrence (local recurrence and/or regional recurrence), and the DSS survival time was calculated from the date of surgery to the date of cancer-related death. Kaplan-Meier analysis (log-rank method) was used to analyse the LRC and DSS rates. The Cox model was used to determine the independent prognostic predictors. All statistical analyses were performed with the help of SPSS 20.0, and *p* < 0.05 was considered to be significant.

## Results

A total of 142 patients (85 male and 57 female) were included for the evaluation. The mean age was 62.7 (range: 34–88) years. Neck metastasis was reported in 27 (19.0%) patients, and extracapsular spread was noted in 8 patients. The mean number of positive nodes was 1.3 (range: 1–3). Clear soft margins were achieved in 100% of the patients. On postoperative pathologic analysis, bone invasion was noted in 32 patients: cortical invasion was noted in 23 patients, and medullary invasion was noted in 9 patients. Perineural invasion was reported in 13 (9.2%) patients, and lymphovascular invasion was reported in 11 (7.7%) patients. Dentate status was described in 113 (79.6%) patients. Tumour differentiation was distributed as follows: well in 81 patients, moderate in 46 patients, and poor in 15 patients. The mean pretreatment neutrophil lymphocyte ratio (NLR) was 2.8 (range: 1.9–8.2) (Table 1).

**Table 1:** General formation of the included patients

Variables	Number (%)
Sex	
Male	85 (59.9%)
Female	57 (40.1%)
Neck lymph node metastasis	27 (19.0%)
Extracapsular spread	8 (5.6%)
Bone invasion	
Cortical invasion	23 (16.2%)
Medullary invasion	9 (6.3%)
Perineural invasion	13 (9.2%)
Lymphovascular invasion	11 (7.7%)
Tumor differentiation	
Well	81 (57.0%)
Moderately	46 (32.4%)
Poorly	15 (10.6%)
Clear soft margin	142(100%)

**Fig. 3:**
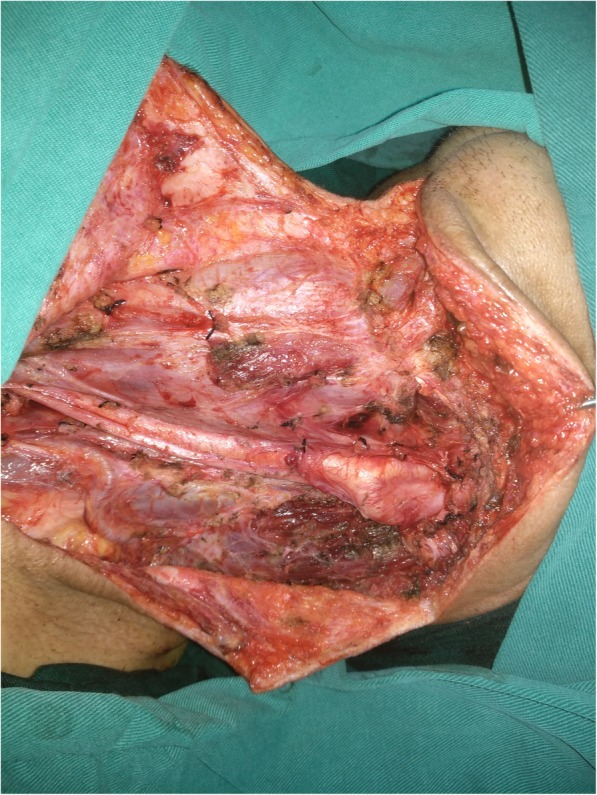
Radical neck dissection for salvage surgery

**Fig. 4:**
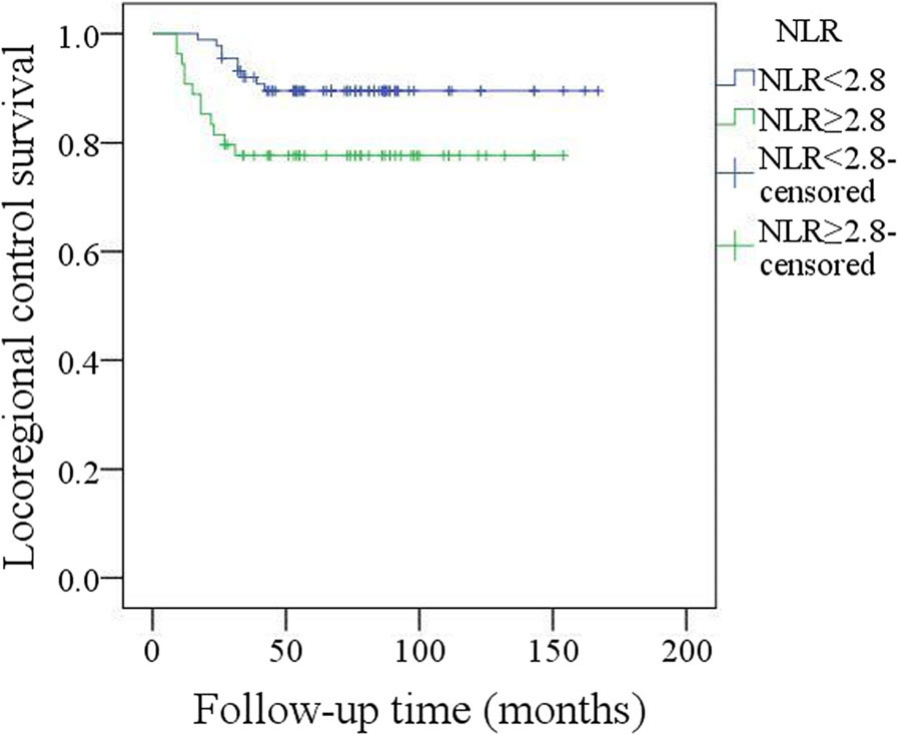
Locoregional control survival in patients with different pretreatment neutrophil lymphocyte ratio (NLR) (*p* = 0.046)

**Fig. 5:**
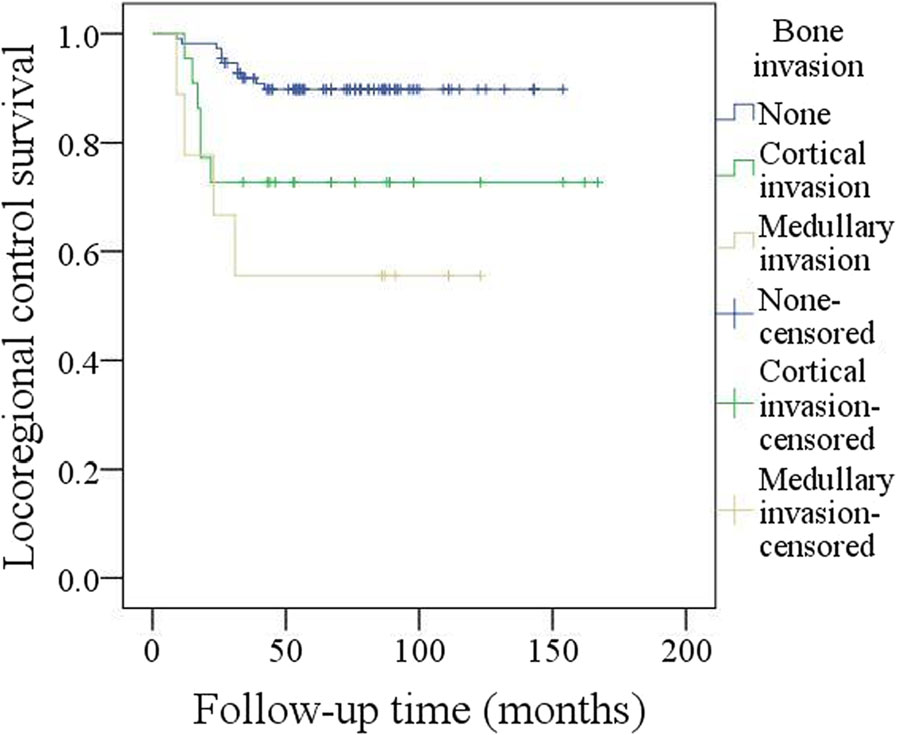
Locoregional control survival in patients with different bone invasion status (*p* = 0.004)

**Fig. 6:**
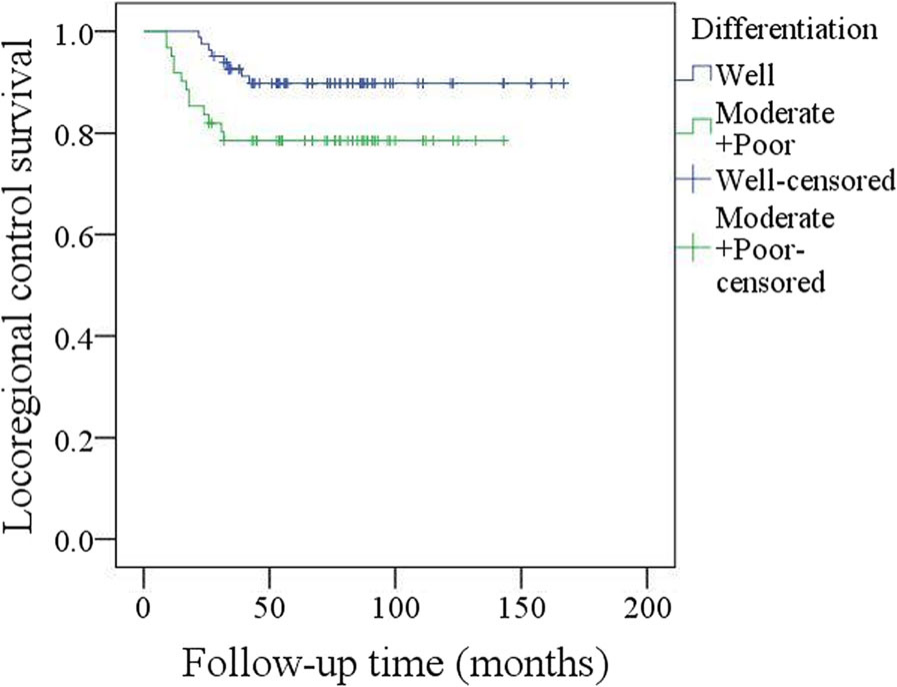
Locoregional control survival in patients with different pathologic tumor differentiation (*p* = 0.039)

Adjuvant radiotherapy was performed in 103 patients, and chemotherapy was performed in 26 patients. After follow-up with a mean time of 69.3 (range: 9–167) months, recurrence occurred in 21 patients: locally in 8 patients and regionally in 13 patients; additionally, there was no distant metastasis. Salvage surgery was successfully performed in 10 patients by segmental mandibulectomy or radical neck dissection (Fig. 3). The 5-year LRC rate was 85%. In the univariate analysis, extent of bone invasion, node metastasis, perineural invasion, poor tumour differentiation, extracapsular spread, and NLR > 2.8 were associated with locoregional recurrence. Further, the Cox model confirmed the independence of NLR (Fig. 4), bone invasion (Fig. 5), and poor tumour differentiation (Fig. 6) in predicting poor LRC (Table 2).

**Table 2:** Univariate and multivariate analysis for locoregional recurrence in patients undergoing marginal mandibulectomy

Variables	Univariate	Cox model	
	Log-rank test	HR(95% CI)	*p*
Age (<62 vs ≥62)	0.268		
Sex	0.456		
Dentate	0.378		
Node stage (N0 vs N+)	< 0.001	2.123(0.936–5.287)	0.099
NLR* (<2.8 vs ≥2.8)	0.046	1.875(1.456–3.512)	0.021
Bone invasion	0.004		
None			
Cortical invasion		1.831 (1.087–3.653)	0.018
Medullary invasion		2.375(1.566–5.997)	0.005
Perineural invasion	0.008	2.872(0.813–8.633)	0.329
Lymphovascular invasion	0.111		
Extracapsular spread	0.010	3.157(0.846–13.662)	0.123
Differentiation (Well vs moderate +poor)	0.039	2.003(1.174–5.088)	0.008
Radiotherapy	0.163		

*: *NLR* neutrophil lymphocyte ratio

**Fig. 7:**
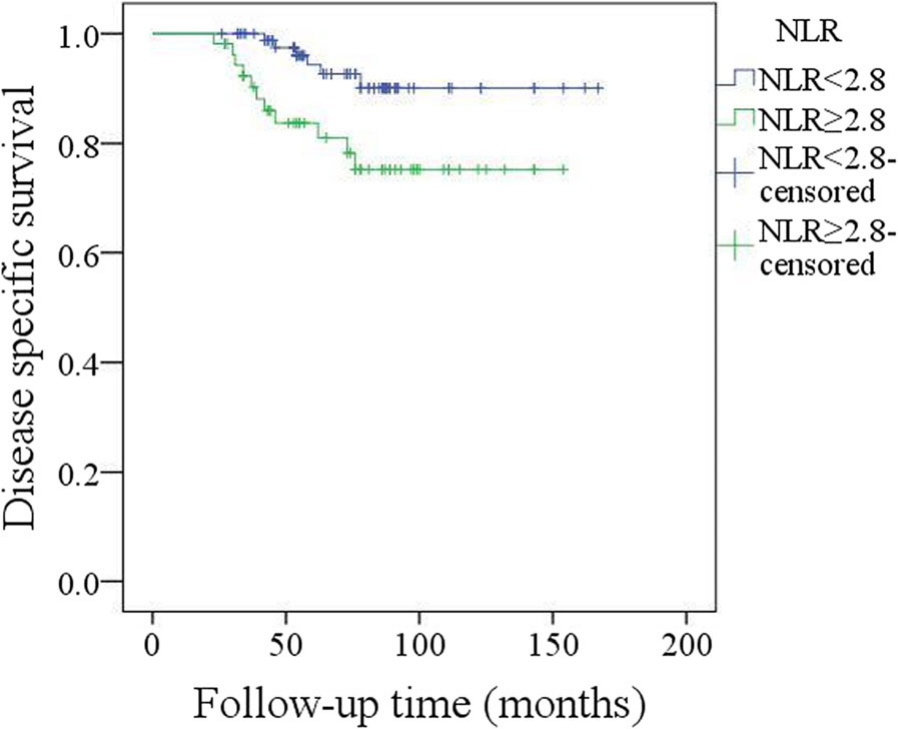
Disease specific survival in patients with different pretreatment neutrophil lymphocyte ratio (NLR) (*p* = 0.029)

**Fig. 8:**
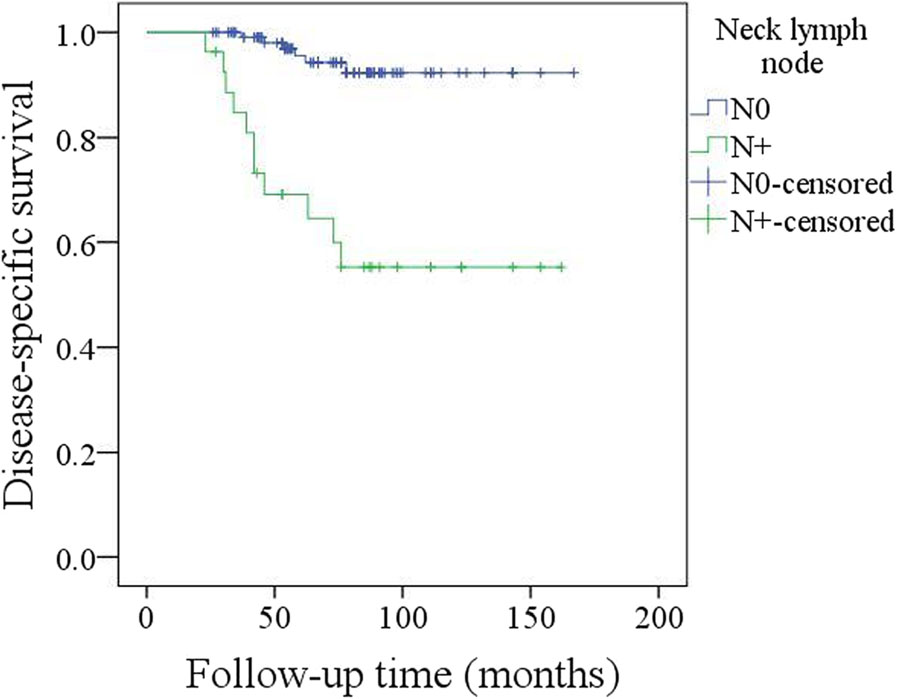
Disease specific survival in patients with different neck lymph node stages (*p* < 0.001)

**Fig. 9:**
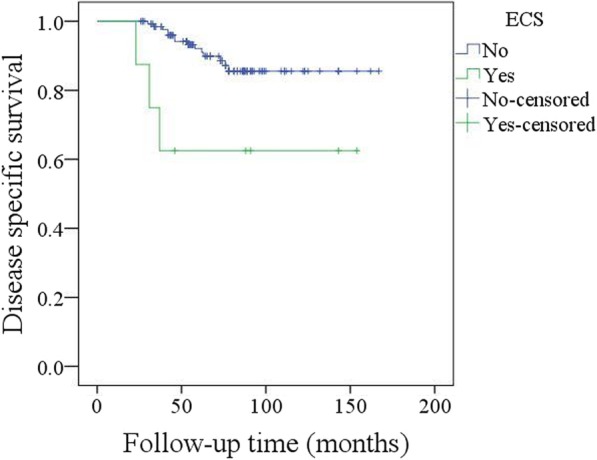
Disease specific survival in patients with different extracapsular spread (ECS) (*p* = 0.004)

A total of 17 patients died of the disease, and the 5-year DSS rate was 88%. In the univariate analysis, node metastasis, lymphovascular invasion, poor tumour differentiation, and extracapsular spread were associated with death. Further, the Cox model confirmed the independence of NLR (Fig. 7), node metastasis (Fig. 8) and extracapsular spread (Fig. 9) in predicting poor DSS (Table 3).

**Table 3:** Univariate and multivariate analysis for cancer-caused death in patients undergoing marginal mandibulectomy

Variables	Univariate	Cox model	
	Log-rank test	HR(95% CI)	*p*
Age (<62 vs ≥62)	0.741		
Sex	0.118		
Dentate	0.412		
Node stage (N0 vs N+)	< 0.001	3.058(1.681–8.229)	< 0.001
NLR* (<2.8 vs ≥2.8)	0.029	1.956(1.067–2.975)	0.018
Bone invasion	0.147		
None			
Cortical invasion			
Medullary invasion			
Perineural invasion	0.315		
Lymphovascular invasion	0.011	1.975(0.561–4.882)	0.265
Extracapsular spread	0.004	2.229(1.349–5.342)	0.001
Differentiation (Well vs moderate +poor)	0.022	2.553(0.876–6.146)	0.143
Radiotherapy	0.655		

*: *NLR* neutrophil lymphocyte ratio

## Discussion

One of the main outcomes in the current study was that bone invasion significantly decreased LRC but not DSS. The prognostic role of bone invasion remains controversial in the literature [7–11]. Shaw et al. [7] described that there was a strong relationship between DSS rate and mandibular invasion. Ogura et al. [8] reported that a high possibility of neck recurrence was associated with bony invasion identified on imaging. However, Patel et al. [9] analysed the oncologic outcome of 111 patients undergoing MM or segmental mandibulectomy, and the authors found that the 5-year local control was similar between the two groups and had no correlation with the extent or presence of bone invasion. Similarly, both Muñoz Guerra et al. [10] and Tankere et al. [11] reported that there was no significant association between the risk of local recurrence and the presence of histologic bone invasion. However, none of the abovementioned studies focused on SCC of the lower gingiva, which might be the most likely disease to involve the mandible. Moreover, in a recent paper, Niu et al. [12] concluded that gingiva SCC of the mandible was not aggressive and had a better prognosis than other sites. On the other hand, regional recurrence was a common treatment failure pattern, but most of above-mentioned studies only focused on local recurrence, the primary endpoint of locoregional control rather than local recurrence might provide more valuable finding. In the current study, we were the first to analyse the extent of bone invasion related to worse locoregional control.

Another interesting finding was that the bone invasion rate was 22.5% in the current study. Petrovic et al. [6] reported that 15.3% of patients undergoing MM had pathologic bone involvement. O’Brien et al. [3] described bone invasion in the marginal resection group in 16% of patients. The difference might be explained by the fact that the two studies enrolled patients with SCC in all oral sub-sites. Gingiva SCC was the most likely to have bone invasion compared with other sites. In a paper published by Okura et al. [13] aiming to analyse the prognosis of SCC of the lower gingiva, the authors found that 58.2% of the patients had mandibular involvement. Similarly, Overholt et al. [14] noted that 41.3% of patients with SCC of the lower gingiva had pathologic bone disease. The difference could be explained by the fact that only early stage gingiva SCC was included in the current study.

Prognosis in the current study was slightly better than that in previous studies. Werning et al. [5] reported that as high as 28% of patients undergoing MM had disease recurrence within two years after initial treatment; in a study performed by Petrovic et al. [6], 12% of patients had neck recurrence, 20.5% of patients had local recurrence, and the 5-year DSS rate was 78.1%; Shaha et al. [15] presented a recurrence rate of 21% at the primary site following MM operation; and Barttlebort et al. [16] reported local recurrence in 25% of patients receiving marginal mandibulectomy. The apparent difference might be due to the positive margin rate. Unlike in other studies, in our study, a clear soft margin was achieved in all patients, there was lower bony involvement, and only early stage disease was included.

Prognostic predictors for head and neck SCC have also been evaluated. The widely accepted risk factors include neck node metastasis, tumour differentiation, perineural invasion, lymphovascular invasion and so on [17–20]. Similar findings were also noted in the current study. Moreover, the prognostic role of the NLR has undergone hot debate. Yu et al. [21] described that an elevated pretreatment NLR in head and neck cancer patients tended to have poorer disease control. Kano et al. [22] found that in patients receiving concurrent chemotherapy for head and neck cancer, there were significant relationships between NLR and cancer sub-site, neck lymph node stage, tumour stage, and disease stage. Further survival analysis indicated the disease-free survival and overall survival were significantly decreased by a high NLR. However, whether there were similar findings in patients with SCC of the lower gingiva remains unknown; the current study was the first to report that a high NLR was associated with worse prognosis.

There were some possible explanations for our interesting finding according to current literature. Firstly, the systemic inflammation and immune system was reflected by the pretreatment NLR, neutrophils are elevated by local and systemic inflammatory, and produce several cytokines and angiogenic factors, then tumour development is promoted by these agents [23]; secondly, haematological markers might be surrogate markers of cancer cachexia, which is associated with poor survival [23, 24]. Thirdly, lymphocytes are related to immune surveillance, and decreased lymphocytes mean that the ability of eliminating cancer cells is inhibited [25, 26]. Therefore, the pretreatment NLR is significantly associated with the prognosis.

The limitations of the current study must be acknowledged. First, this was a retrospective study; thus, there is inherent bias that might have decreased the statistical power. Second, the sample size was relatively small; thus, more large prospective studies are needed to clarify the conclusion.

## Conclusions

In summary, the oncologic outcome in patients undergoing MM for cT1-2 N0 SCC of the lower gingiva was favourable; furthermore, bone invasion was not uncommon, but it significantly decreased prognosis in patients undergoing MM.

## Data Availability

All data generated or analysed during this study are included in this published article. The primary data can be obtained from the corresponding author.

## Abbreviations

DSS: Disease-specific survival
LRC: Locoregional control
MM: Marginal mandibulectomy
NLR: Neutrophil lymphocyte ratio
SCC: Squamous cell carcinoma
